# Peer Review of “Comparison Between Male and Female Survivors of Sexual Abuse and Assault in Relation to Age at Admission to Therapy, Age of Onset, and Age at Last Sexual Assault: Retrospective Observational Study”

**DOI:** 10.2196/34623

**Published:** 2021-11-26

**Authors:** Petroula Laiou

**Affiliations:** 1 Department of Biostatistics and Health Informatics Institute of Psychiatry, Psychology and Neuroscience King's College London London United Kingdom

**Keywords:** sexual abuse, sexual assault, age of onset, sex, gender, age, therapy, abuse, assault, mental health, victim, childhood, children, gender disparity, violence

*This is a peer-review report submitted for the paper “Comparison Between Male and Female Survivors of Sexual Abuse and Assault in Relation to Age at Admission to Therapy, Age of Onset, and Age at Last Sexual Assault: Retrospective Observational Study.”*

## Round 1 Review

### General Comments

This multicenter study [1] explores differences between male and female victims who were sexually abused or assaulted and sought therapy. The differences are investigated with regard to the age of the first and last assault as well as the age at which the victims entered therapy. Although there is a high imbalance between the female (2901) and male (401) groups, the author provides the data distributions, detailed statistical metrics, and explanations of the studied cohorts that allow the reader to have a good understanding of the investigated cohorts.

### Specific Comments

Overall, it is a well-written and clear paper. The author makes an extensive introduction and provides a detailed description of the study results. The quality of the paper could be improved by considering the following aspects:

#### Major Comments

1. The sections *Limitations* and *Conclusions* are given in bullet format. This formatting should be converted to a paragraph-like formatting. Additionally, the *Limitations* section should be placed before the *Conclusions* section and not after, as it is now.

2. In the *Discussion* section, more text is needed regarding the comparison of the current study with previous studies.

3. When a linear relationship is reported in the paper (Figure 5), it is better for it to be accompanied by a statistical metric, such as the Spearman correlation.

#### Minor Comments

1. There are few typos in the paper.

a. *Introduction* section (second paragraph, first sentence)—please correct the year. Obviously, it is not 2104.

b. Other minor typos; for example, please replace the phrase “first the sexual assault” with “the first sexual assault” (second paragraph before the end of the *Introduction*) as well as “Cleary” with “Clearly.”

2. Suggestion for the author: In future studies that deal with highly imbalanced data sets, the author could consider using bootstrapping methods. For example, in the current study, the number of victims in the males group was 401 and that in the females group was 2901. If we assume that we study the differences in the two groups with regard to the age of the first assault, we could do the following: In the male group, we compute the mean value of the age of the first assault using all 401 victims. In the females group, we randomly take a subcohort of 401 victims multiple times (eg, 1000). In every random selection, we compute the mean value of the age of assault; hence, at the end, for the female group, we will have a distribution of 1000 values. Afterward, from the distribution of these 1000 values, we can compute confidence intervals, medians, etc. In this way, the number between the female and male group is the same, and therefore the results between the two groups are more comparable.
